# Supplementary material for: Exploring Lignin Biosynthesis Genes in Rice: Evolution, Function, and Expression
Source: Int J Mol Sci. 2024 Sep 17;25(18):10001. doi: 10.3390/ijms251810001 (PMC11432410; doi:10.3390/ijms251810001)
Supplement: Supplementary file 1 [file ijms-25-10001-s001.zip › ijms-3185650-supplementary.pdf]

Supplementary materials

Table S1. Basic information of 90 lignin-related genes identified in rice.

No.	Genes	TIGR Loci	Probsets[a]	Protein characteristics		Number of AA
				Pred Hel[b]	Domains	
1	OsPAL1	LOC_Os02g41630	Os.9330.4.S1_x_at	NONE	PAL-HAL	701
2	OsPAL2	LOC_Os04g43760	Os.37893.1.S1_at	NONE	PAL-HAL, Lyase_I_like	340
3	OsPAL3	LOC_Os11g48110	N/A	1	PAL-HAL	700
4	OsPAL4	LOC_Os12g33610	Os.27728.1.S1_at	1	PAL-HAL	680
5	OsPAL5	LOC_Os04g43800	Os.52536.1.S1_at	NONE	PAL-HAL	714
6	OsPAL6	LOC_Os02g41650	Os.28397.1.S3_at	NONE	PAL-HAL	718
7	OsPAL7	LOC_Os05g35290	N/A	NONE	PAL-HAL	716
8	OsPAL8	LOC_Os02g41680	Os.25687.1.S1_x_at	NONE	PAL-HAL	713
9	OsPAL9	LOC_Os02g41670	Os.10930.1.S1_at	NONE	PAL-HAL	713
10	OsC4H1	LOC_Os01g60450	OsAffx.2373.1.S1_x_at	2	p450	481
11	OsC4H2	LOC_Os05g25640	Os.53884.1.S1_at	1	p450	500
12	OsC4H3	LOC_Os02g26770	N/A	3	p450	533
13	OsC4H4	LOC_Os02g26810	Os.11417.1.S1_at	3	p450	533
14	Os4CL1	LOC_Os08g14760	Os.52921.1.S1_at	NONE	LuxE	564
15	Os4CL2	LOC_Os02g46970	Os.4377.1.S1_at	1	LuxE	569
16	Os4CL3	LOC_Os02g08100	Os.10669.1.S1_at	NONE	LuxE	554
17	Os4CL4	LOC_Os06g44620	Os.8020.2.S1_at	NONE	LuxE	445
18	Os4CL5	LOC_Os08g34790	Os.19375.1.S1_at	NONE	LuxE	539
19	Os4CLL1	LOC_Os03g05780	Os.11459.1.S1_at	1	LuxE	552
20	Os4CLL2	LOC_Os10g42800	OsAffx.30755.1.S1_x_at	NONE	LuxE	564
21	Os4CLL3	LOC_Os08g04770	OsAffx.29036.1.S1_at	NONE	LuxE	591
22	Os4CLL4	LOC_Os03g04000	Os.18327.1.S1_at	2	LuxE	552
23	Os4CLL5	LOC_Os01g67530	Os.5174.1.S1_at	NONE	LuxE	442
24	Os4CLL6	LOC_Os01g67540	OsAffx.21790.1.S1_at	3	LuxE	598
25	Os4CLL7	LOC_Os07g17970	Os.15398.1.S1_at	NONE	LuxE	483
26	Os4CLL8	LOC_Os07g44560	OsAffx.5636.1.S1_at	NONE	LuxE	609
27	Os4CLL9	LOC_Os04g24530	OsAffx.13955.1.S1_x_at	1	LuxE	522
28	OsHCT1	LOC_Os04g42250	Os.12225.1.S1_at	NONE	Transferase	442
29	OsHCT2	LOC_Os02g39850	Os.7870.1.S1_at	NONE	Transferase	442
30	OsHCT3	LOC_Os06g08580	Os.15299.1.S1_x_at	NONE	Transferase	445
31	OsHCT4	LOC_Os06g08640	OsAffx.27513.1.S1_s_at	NONE	Transferase	433
32	OsHCT5	LOC_Os09g25460	Os.37941.1.S1_at	NONE	Transferase	440
33	OsHCT6	LOC_Os08g43040	Os.15257.1.S1_at	1	Transferase	442
34	OsHCT7	LOC_Os11g07960	Os.10765.1.S1_at	NONE	Transferase	447
35	OsHCT8	LOC_Os08g10420	OsAffx.5777.1.S1_at	NONE	Transferase	458
36	OsC3H	LOC_Os05g41440	Os.5391.1.S1_at	NONE	p450	362
37	OsCCoAOMT1	LOC_Os06g06980	Os.4198.1.S1_x_at	NONE	AdoMet_Mtases	260
38	OsCCoAOMT2	LOC_Os09g30360	Os.4830.1.S1_x_at	NONE	AdoMet_MTases	258
39	OsCCoAOMT3	LOC_Os08g38910	Os.7348.2.S1_x_at	NONE	AdoMet_MTases	203
40	OsCCoAOMT4	LOC_Os08g38920	N/A	NONE	AdoMet_Mtases	234
41	OsCCoAOMT5	LOC_Os08g38900	Os.4244.1.S1_at	NONE	AdoMet_MTases	190
42	OsCCoAOMT6	LOC_Os08g05790	Os.24052.1.S1_x_at	NONE	AdoMet_MTases	283
43	OsCCR1	LOC_Os09g25150	Os.9685.1.S1_a_at	1	FR_SDR_e	246
44	OsCCR2	LOC_Os08g34280	Os.8544.1.S1_at	NONE	FR_SDR_e	361
45	OsCCR3	LOC_Os08g17500	Os.54671.1.S1_at	NONE	FR_SDR_e	342

46	OsCCR4	LOC_Os02g08420	Os.12948.1.S1_at	1	FR_SDR_e	344
47	OsCCR5	LOC_Os09g04050	Os.53814.1.S1_at	NONE	FR_SDR_e	347
48	OsCCR6	LOC_Os01g18110	Os.45894.1.S1_x_at	NONE	FR_SDR_e	326
49	OsCCR7	LOC_Os01g18120	Os.20420.1.S1_at	NONE	FR_SDR_e	328
50	OsCCR8	LOC_Os09g08720	OsAffx.29770.1.S1_at	NONE	FR_SDR_e	324
51	OsCCR9	LOC_Os02g56700	Os.20154.1.S1_at	NONE	FR_SDR_e	339
52	OsCCR10	LOC_Os02g56690	OsAffx.24898.2.S1_at	NONE	FR_SDR_e	354
53	OsCCR11	LOC_Os02g56720	Os.55206.1.A1_at	NONE	FR_SDR_e	334
54	OsCCR12	LOC_Os02g56460	Os.6757.1.S1_at	NONE	FR_SDR_e	338
55	OsCCR13	LOC_Os02g56680	N/A	NONE	FR_SDR_e	337
56	OsCCRL1	LOC_Os06g41840	Os.6207.1.S1_s_at	NONE	FR_SDR_e	187
57	OsCCRL2	LOC_Os06g41810	Os.15971.1.S1_at	NONE	FR_SDR_e	321
58	OsCCRL3	LOC_Os09g31502	Os.51220.1.S1_x_at	NONE	FR_SDR_e	281
59	OsCCRL4	LOC_Os09g31490	Os.15746.1.S1_at	NONE	FR_SDR_e	343
60	OsCCRL5	LOC_Os09g31506	N/A	NONE	FR_SDR_e	220
61	OsCCRL6	LOC_Os09g31514	Os.17520.1.S1_at	NONE	FR_SDR_e	249
62	OsCCRL7	LOC_Os03g60380	Os.24712.1.A1_s_at	NONE	FR_SDR_e	334
63	OsCCRL8	LOC_Os01g61230	Os.42521.1.S1_at	NONE	FR_SDR_e	326
64	OsCCRL9	LOC_Os01g74660	Os.5806.1.S1_at	NONE	FR_SDR_e	327
65	OsCCRL10	LOC_Os08g40440	OsAffx.17435.1.S1_s_at	NONE	FR_SDR_e	295
66	OsCCRL11	LOC_Os01g34480	Os.11827.1.S1_a_at	NONE	FR_SDR_e	284
67	OsCCRL12	LOC_Os05g50250	Os.51338.1.S1_at	NONE	FR_SDR_e	379
68	OsCCRL13	LOC_Os01g45200	Os.11247.1.S1_at	NONE	FR_SDR_e	363
69	OsF5H1	LOC_Os10g36848	Os.9727.1.S1_at	NONE	p450	530
70	OsF5H2	LOC_Os03g02180	OsAffx.12708.1.S1_x_at	1	p450	519
71	OsF5H3	LOC_Os06g24180	OsAffx.4924.1.S1_at	NONE	p450	529
72	OsCOMT	LOC_Os08g06100	Os.11202.1.S1_at	NONE	Methyltransf_2, Dimerisation	368
73	OsCOMTL1	LOC_Os12g13810	OsAffx.19658.1.S1_at	NONE	Methyltransf_2	262
74	OsCOMTL2	LOC_Os12g10140	OsAffx.31676.1.S1_at	NONE	Methyltransf_2	250
75	OsCOMTL3	LOC_Os04g01470	Os.23187.1.S1_at	NONE	Methyltransf_2, Dimerisation	357
76	OsCOMTL4	LOC_Os04g09654	Os.10277.1.S1_at	NONE	Methyltransf_2, Dimerisation	258
77	OsCOMTL5	LOC_Os04g09680	N/A	NONE	Methyltransf_2	171
78	OsCOMTL6	LOC_Os04g09604	Os.26569.1.S1_at	NONE	Methyltransf_2	378
79	OsCOMTL7	LOC_Os02g57760	Os.54406.1.S1_at	NONE	Methyltransf_2	365
80	OsCAD1	LOC_Os10g11810	Os.7543.1.S1_at	NONE	CAD1	297
81	OsCAD2	LOC_Os02g09490	Os.8684.1.S1_a_at	NONE	CAD1	363
82	OsCAD3	LOC_Os10g29470	Os.7496.1.S1_a_at	NONE	CAD1	366
83	OsCAD4	LOC_Os11g40690	N/A	NONE	CAD1	343
84	OsCAD5	LOC_Os08g16910	N/A	NONE	CAD1	332
85	OsCAD6	LOC_Os04g15920	Os.6089.1.S1_at	NONE	CAD1	360
86	OsCAD7	LOC_Os04g52280	Os.32904.1.S1_at	NONE	CAD1	379
87	OsCAD8A	LOC_Os09g23530	Os.5983.1.S1_at	NONE	CAD1	359
88	OsCAD8B	LOC_Os09g23540	Os.37839.1.S1_at	NONE	CAD1	436
89	OsCAD8C	LOC_Os09g23550	Os.6862.2.A1_at	NONE	CAD1	439
90	OsCAD8D	LOC_Os09g23560	N/A	NONE	CAD1	362

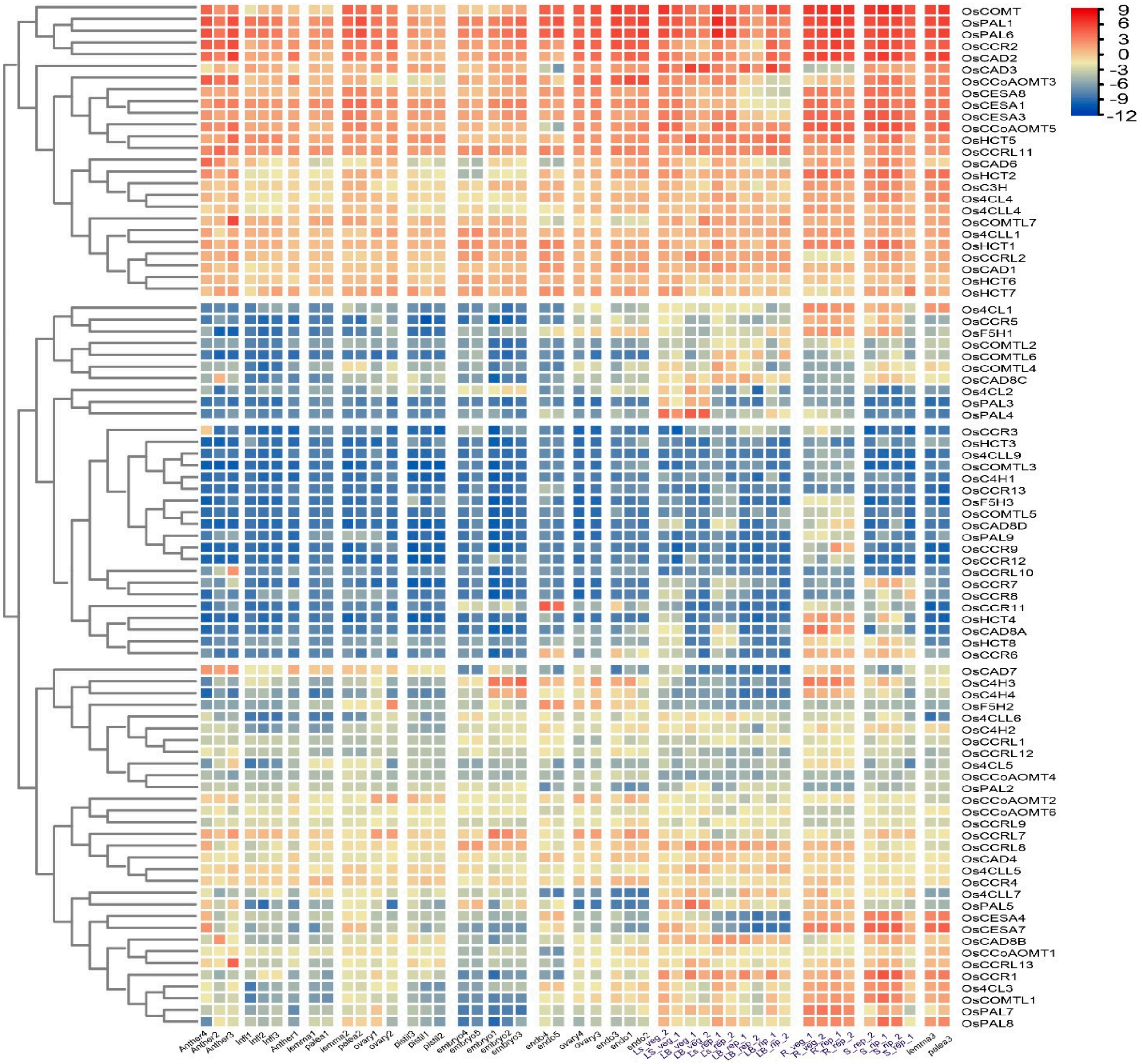

Figure S1. Co-expression clustering of rice lignin gene expression profiles in Nipponbare cultivar at different growth stages. Normalized count values data were downloaded from the RiceXpro database. The scale at the extreme top right represents the level of gene expression as red, yellow, and blue exhibit relatively high, medium, and low expressions, respectively. The tissue samples in the lower part of the figure in blue represent vegetative while in black show reproductive tissue samples.

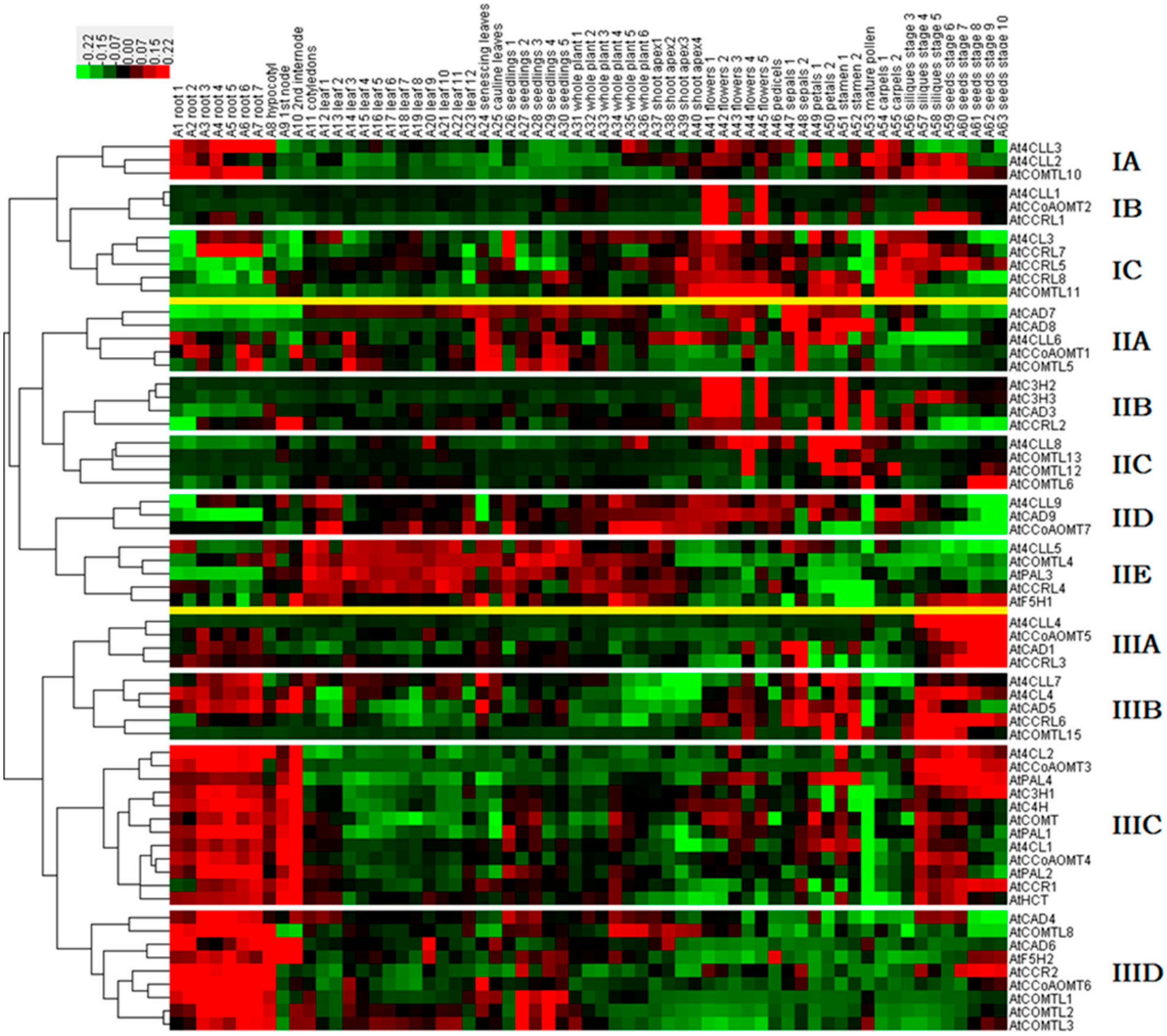

Figure S2. The lignin-related genes co-expression profiling (Log transformed) in *Arabidopsis*. The color scale representing the relative signal values is shown above (green refers to low expression; black refers to medium expression; red refers to high expression).

Table S2. Conserved amino acid motifs in the protein sequences of lignin biosynthesis genes.

MOTIF	WIDTH	BEST POSSIBLE MATCH
1	65	LPMLVYVSREKRPGYNHHKKAGAMNALVRVSAVMTNAPFMLNLDCDHYINNSQAIREAMCFMMDP
2	75	STVDPMKEPPLYTANTILSILAVDYPVDKVSCYVSDDGGAMLTFEAMAETAEFARKWVPFCKKHCIEPRAPEWYF
3	81	FFGFNGTAGVWRIKAINESGGWMDRTTVEDMDIAVRAHLKGWKFIYLNDVKCKCELPSTYKAYRHQQHRWHCGPMNLFRKC
4	80	VISCWYEDKTEWGKEIGWIYGSVTEDIVTGFRMHCRGWRSVYCMPKRDAFKGTAPINLTDRLHQVLRWATGSVEIFFSHH
5	62	DKVCYVQFPQRFDGIDPHDRYANHNTVFFDINMKGLDGIQGPMYVGTGCMFRRQALYGYDPP
6	80	NLKEGMECDYVKQCEYVAIFDADFQPEPDFLKRTVPHFKHNPEIGLVQARWSFVNKDECLMTRMQNMNLCYHFKVEQQVN
7	47	MTGMLEMKWSGITIEDWWRNEQFWMIGGTSAHLFAVFQGLLKVLTGI
8	115	IIINKKISFWKKIHLIYSFFLVRKIIAPFYTFTFYCIIIPMTMFVPEVEIPIWGVCYIPTTMTILNIIPNPKSFHFIIFWILFENTMSMHRMNAMISGLFQLGSANE WVVTKKSG
9	32	EIWFAFSWILDQFPKWCPINRETYLDRLAERY
10	41	LYGRRLKFLQRMAYINMTIYPFTSIFLLAYCTLPAICLLTG
11	41	MVLVQIPMCNEKEVYQQSIGAVCNLDWPRDRFIVQVLDDST
12	29	LFFNFWVIVHLYPFAKGLMGRQNRTPTIV
13	57	MDEARQPLSRKYPIPSSIIHPYRMLIIIRLVVLGFFFHWRITHPNRDAMWLWTMSVI
14	44	KIDYYKDKVQPEFVKDRRRMKREYEEFKVRINALPAKIQKRPEE
15	45	TKQTAADEDDEFAELYTFKWTTLLIPPTTIMMVNIIGIVAGVSKA
16	70	NGQVCQICGDDVGTTPDGEPFVACNECGFPVCRPCYEYERREGTQCCPQCKTRYKRHKGCPRVPGDEEED
17	52	KGPKATWMADGTHWPGTWIEPSENHQKGDHAGIVQVMLNHPSHKPQLGMPAS
18	29	GWIMQDGTPWPGNNTRDHPGMIQVFLGHS
19	29	VEEECEKWQQKGVNIKYEHRNNRKGYKAG
20	41	KKKHNRIYKKELALSFFLLTCACYDLLYHQGIHFYFLLFQG
21	21	VVWSILLASIFSLLWVRIDPF
22	159	FVCTADPHAEPPSLVISTILSVMAYNYPSEKISVYLSDDGGSILTFYALWEASIFAKKWLPFCKRYNIEPRSPAAYFSESEGHHNLCGPKEWAFIKNLYEE MRERIDSAVMSGKIPEEMKLKHKGFDEWNSDFTSKNHQPIVQILIDGKNQNAVDDDGN
23	21	KKFGQSSVFIASTLMEQNQER
24	29	WMQVRADYVAPPLQFLVWACMVMFMMQSA
25	56	IAFVQYPQRFDNVDPNDIYGNHNNVFFDCTMLGLDGLQGCIYIGTGCFHRREALCG
26	32	EIWFAFSWILDQFPKWCPINRETYLDRLAERY
27	44	KIDYYKDKVQPEFVKDRRRMKREYEEFKVRINALPAKIQKRPEE
28	29	GWIMQDGTPWPGNNTRDHPGMIQVFLGHS
29	45	TKQTAADEDDEFAELYTFKWTTLLIPPTTIMMVNIIGIVAGVSKA
30	21	VVWSILLASIFSLLWVRIDPF

Table S3. The lignin monomer composition and total lignin contents in five genotypes at six growth stages (μmol/g dry matter).

Sample	Stage	H	G	S	H+G+S	S/G	H/G	S/H
NPB	I	37.98±1.89	48.00±1.89	26.73±2.42	112.72±4.73	0.56	0.79	0.70
	II	47.77±2.05	71.87±2.38	36.08±0.67	155.73±3.74	0.50	0.66	0.76
	III	67.33±1.00	85.16±0.89	56.96±1.86	209.46±3.64	0.67	0.79	0.85
	IV	67.38±0.57	104.30±0.54	62.06±0.89	233.74±1.82	0.60	0.65	0.92
	V	86.71±3.23	119.69±3.57	79.13±1.79	285.53±4.63	0.66	0.72	0.91
	VI	129.56±2.14	145.30±2.55	79.77±1.00	354.62±5.61	0.55	0.89	0.62
C6	I	27.99**±0.48	36.69**±1.92	13.29*±0.09	77.97**±2.36	0.36	0.76	0.47
	II	49.98*±2.16	79.62**±2.00	35.44±0.71	165.03**±4.61	0.45	0.63	0.71
	III	67.11±1.99	99.90**±1.15	41.21**±0.70	208.23±3.04	0.41	0.67	0.61
	IV	66.45±0.98	121.20**±1.34	53.56**±1.78	241.20**±2.15	0.44	0.55	0.81
	V	91.20±1.12	146.68**±2.77	58.27**±0.83	296.16*±3.87	0.40	0.62	0.64
	VI	108.75**±2.61	174.86**±2.91	66.42**±0.97	350.03±6.31	0.38	0.62	0.61
C15	I	33.04*±1.14	44.27**±1.43	22.10±0.43	99.41**±2.56	0.50	0.75	0.67
	II	52.11**±2.09	57.82**±0.98	30.45*±1.56	140.38**±4.57	0.53	0.90	0.58
	III	64.38±1.50	75.59**±1.92	40.67**±0.24	180.64**±2.09	0.54	0.85	0.63
	IV	74.05*±1.67	90.91**±1.08	53.58**±1.41	218.54*±4.15	0.59	0.81	0.72
	V	86.20±2.79	107.71*±1.00	68.72*±1.05	262.64**±4.68	0.64	0.80	0.80
	VI	138.46*±3.55	112.84**±1.23	106.64**±1.72	357.95±3.46	0.95	1.23	0.77
C17	I	39.41*±2.12	48.00±0.42	23.67±1.11	111.08±3.29	0.49	0.82	0.60
	II	59.32*±2.22	83.22**±1.24	39.42±1.49	181.96**±3.98	0.47	0.71	0.66
	III	62.88±3.13	104.49**±1.31	62.49±2.19	229.86**±2.55	0.60	0.60	0.99
	IV	89.11**±1.72	101.66±1.45	64.37±2.59	255.13**±3.26	0.63	0.88	0.72
	V	108.17**±2.60	113.05±0.52	75.54±1.70	296.75*±4.42	0.67	0.96	0.70
	VI	152.56**±1.71	127.46**±1.82	102.67**±2.23	382.69**±4.51	0.81	1.20	0.67
Y102	I	36.68±2.73	31.94**±1.31	18.73*±1.83	87.35**±4.00	0.59	1.15	0.51
	II	56.84**±2.75	51.97*±2.67	34.48±2.06	143.30*±1.54	0.66	1.09	0.61
	III	59.60*±3.72	63.10**±0.47	41.37**±0.72	164.07**±3.86	0.66	0.94	0.69
	IV	70.78±1.99	73.43**±1.52	38.20**±1.24	182.42**±3.77	0.52	0.96	0.54
	V	101.31*±0.66	95.38**±0.92	72.21**±1.88	268.90**±2.25	0.76	1.06	0.71
	VI	132.48*±1.30	126.54**±1.36	86.90*±2.57	345.91±2.15	0.69	1.05	0.66

Note; NPB was used as the control group, while the four mutants were used as comparison groups. * and ** A significant difference by t-test at $p < 0.05$ and 0.01 (n = 3); and Stage of samples (I refers to the 2nd internode length is 0-2 cm; II refers to the length is 3-5 cm; III refers to the length is 6-8 cm; IV refers to the length is 10-12 cm; V refers to the length is greater than 13 cm; VI refers to mature internode). The deviation around the mean values is indicated by the (±) sign.

Table S4. The monomer composition of total lignin increased between two consecutive stages (μmol/g dry matter).

Genotype	Stages	H	G	S	H+G+S
	I-II&	9.79**±0.27	23.87**±0.62	9.35±1.88	43.01**±1.65
	II-III	19.56**±1.06	13.29*±1.67	20.88*±1.34	53.73**±1.47
NPB	III-IV	0.04±0.50	19.14**±0.35	5.11±0.99	24.29*±2.03
	IV-V	19.33*±2.69	15.38±3.10	17.07**±0.91	51.78*±3.10
	V-VI	42.85**±1.48	25.61**±1.02	0.63±1.21	69.09**±3.59
	I-II	21.98*±1.71	42.93***±0.34	22.15**±0.64	87.07**±2.54
	II-III	17.14*±0.94	20.28*±1.21	5.78***±0.07	43.19**±2.02
C6	III-IV	-0.67±1.08	21.3***±0.18	12.34*±1.09	32.98**±1.78
	IV-V	24.76***±0.15	25.48*±2.06	4.71±1.41	54.95**±2.29
	V-VI	17.55*±1.63	28.17**±1.35	8.15*±1.31	53.87**±2.85
	I-II	19.06*±1.13	13.56**±0.57	8.35*±1.16	40.97*±2.32
	II-III	12.27**±0.60	17.77*±1.41	10.22*±1.32	40.26*±2.50
C15	III-IV	9.67*±0.86	15.32*±0.85	12.91*±1.23	37.90*±2.59
	IV-V	12.15*±1.63	16.8**±0.84	15.14*±1.13	44.09*±3.15
	V-VI	52.26**±1.17	5.13*±0.52	37.92**±0.72	95.31***±1.32
	I-II	19.9**±0.40	35.22**±0.87	15.76*±0.94	70.88**±2.20
	II-III	3.57±1.18	21.27***±0.15	23.07**±0.72	47.90**±2.23
C17	III-IV	26.23*±2.28	-2.83±0.72	1.87±0.77	25.27*±1.68
	IV-V	19.06*±1.77	11.39*±1.19	11.17*±0.89	41.62**±1.23
	V-VI	44.39**±1.19	14.41*±1.73	27.14*±1.57	85.94***±0.21
	I-II	20.16*±1.73	20.03*±1.77	15.76**±0.70	55.94**±2.69
	II-III	2.75±2.13	11.13±2.32	6.88±1.36	20.77*±2.90
Y102	III-IV	11.19±2.21	10.33*±1.21	-3.16±0.57	18.35**±0.43
	IV-V	30.53**±1.36	21.94**±0.71	34.01**±0.96	86.48**±1.64
	V-VI	31.16**±0.65	31.16***±0.48	14.68*±0.95	77.01***±0.58

Note: *, ** and *** A significant difference by *t*-test at $p < 0.01$, 0.001 and 0.0001 (n = 3); Stage of samples (I-II refers to the 2nd internode length is 0-2 to 3-5 cm; II-III refers to the length is 3-5 to 6-8 cm; III-IV refers to the size is 6-8 to 10-12 cm; IV-V refers to the length is 10-12 to greater than 13 cm; V-VI refers to the length is greater than 13 cm to mature). The deviation around the mean values is indicated by the (±) sign.

Table S5. Twenty-seven genes with high expression levels from the CREP database in the stem were selected for qPCR.

No.	Genes	TIGR Loci	No.	Genes	TIGR Loci
1	Os4CL1	LOC_Os08g14760	16	OsCOMT	LOC_Os08g06100
2	Os4CL3	LOC_Os02g08100	17	OsCOMTL4	LOC_Os04g09654
3	Os4CL5	LOC_Os08g34790	18	OsCOMTL6	LOC_Os04g09604
4	OsC3H	LOC_Os05g41440	19	OsF5H1	LOC_Os10g36848
5	OsC4H2	LOC_Os05g25640	20	OsF5H2	LOC_Os03g02180
6	OsCAD1	LOC_Os10g11810	21	OsF5H3	LOC_Os06g24180
7	OsCAD2	LOC_Os02g09490	22	OsHCT1	LOC_Os04g42250
8	OsCAD8A	LOC_Os09g23530	23	OsHCT2	LOC_Os02g39850
9	OsCAD8C	LOC_Os09g23550	24	OsHCT3	LOC_Os06g08580
10	OsCCoAOMT1	LOC_Os06g06980	25	OsPAL2	LOC_Os04g43760
11	OsCCoAOMT5	LOC_Os08g38900	26	OsPAL8	LOC_Os02g41680
12	OsCCR1	LOC_Os09g25150	27	OsPAL9	LOC_Os02g41670
13	OsCCR2	LOC_Os08g34280			
14	OsCCR3	LOC_Os08g17500			
15	OsCCR8	LOC_Os09g08720			

Table S6. The primers of 27 genes for qRT-PCR.

Gene name	PCR primer
**4CL1-F**	5' GCCCTTCTCATCACCCATCCAA 3'
**4CL1-R**	5' GCGACTGGTATTTCTCCGATTT 3'
**4CL3-F**	5' CCTGAGGCGACCAAGAACAC 3'
**4CL3-R**	5' CGGTGATTTCTGAGCCTTCTG 3'
**4CL5-F**	5' GGCCGGTGCTATCAATGTG 3'
**4CL5-R**	5' GGGTTGTTCAGGTATCCTTTCAT 3'
**C3H-F**	5' CAGTTATTGGGCTTCTATGGGAC 3'
**C3H-R**	5' GACGGCGTTCAGGTAAGGG 3'
**C4H2-F**	5' TGAACCACCCGAGCATCC 3'
**C4H2-R**	5' CCGCAGCGTCTCCTTCAC 3'
**CAD1-F**	5' TGAAAGTTGGTGGTGTAATGGC 3'
**CAD1-R**	5' AAGCGAAATCTGACATCCCG 3'
**CAD2-F**	5' GACTCGCTGGACTACATCATCG 3'
**CAD2-R**	5' AGTTGAGCACCTCCTCCGTCT 3'
**CAD8A-F**	5' AAGGGTTCAGGAGGAAGACGA 3'
**CAD8A-R**	5' CGTTCTTGATGGTGTGCAGGT 3'
**CAD8C-F**	5' GGAGGGCTCAGAAGGACGAT 3'

**CAD8C-R**	5' GCACGACGCGACGAAGTAG 3'
**CCoAOMT1-F**	5' GCTGGTGGAGGAGGAGGG 3'
**CCoAOMT1-R**	5' TCATCAGCCGCTCGTGGT 3'
**CCoAOMT5-F**	5' CTGGGGATGCTGCTGAAGAT 3'
**CCoAOMT5-R**	5' CCACCACCTTCCCGTCCT 3'
**CCR1-F**	5' CCAAGAAGTACGCCAATGCC 3'
**CCR1-R**	5' GCTGCTTCCGTGGGTTCA 3'
**CCR2-F**	5' CTGCTGGAGCGACCTTGACT 3'
**CCR2-R**	5' TTGAGGATGTGGGCGACG 3'
**CCR3-F**	5' GGACACCGGAAACTGGTACTG 3'
**CCR3-R**	5' AGCCGTCCAGGTACTTGAGC 3'
**CCR8-F**	5' GGAATGCTGTCGCTGGGTAT 3'
**CCR8-R**	5' TCTGCTTGCTGTCTTCACACTTG 3'
**COMT-F**	5' AGGGAGCAGGGGGTGTTC 3'
**COMT-R**	5' CAGGCGTTGGCGTAGATGTA 3'
**COMTL4-F**	5' ATTGTTGTTGATATTGTTCTCCCTG 3'
**COMTL4-R**	5' TCCTGCTCTGTCCTTATCTTTCC 3'
**COMTL6-F**	5' CAGCACGATACCCGTTCCA 3'
**COMTL6-R**	5' GTTCGCAAGGCACCACTGA 3'
**F5H1-F**	5' GCGATGGCGGAGATGATG 3'
**F5H1-R**	5' GATGACGCAGCGGAGGAA 3'
**F5H2-F**	5' ATGGACCCGTGGCTTGTTC 3'
**F5H2-R**	5' GCTCGACACCACCACCATG 3'
**F5H3-F**	5' GTGGAGTTCAAGGGTGGGG 3'
**F5H3-R**	5' AGAGCAACGGGCAGGACA 3'
**HCT1-F**	5' CATCTCCTCCTTCTCGCTCCT 3'
**HCT1-R**	5' TCGTAGGAGAGTGCGGTCAAT 3'
**HCT2-F**	5' TGGTTCCGTTCTACCCGATG 3'
**HCT2-R**	5' GGATGGGGAAGGAGGAGATG 3'
**HCT3-F**	5' CTCGGCAACGCCATCTTC 3'
**HCT3-R**	5' TCCAGGTGGTCCAGCAGC 3'
**PAL2-F**	5' TCTACAACAACGGGCTCACG 3'
**PAL2-R**	5' GCAACCAGGTAGGTGGAGGA 3'
**PAL8-F**	5' GCCTGCCATCCAACCTGTC 3'
**PAL8-R**	5' TTGCCTCGTCGGTCTTCCT 3'
**PAL9-F**	5' CTGGTCCCGCTCTCCTACAT 3'
**PAL9-R**	5' CTCGGCAAGGACAGCAAGA 3'

Table S7. The qRT-PCR gene expressions of 27 genes in Nipponbare (NPB).

Genes	I	II	III	IV	V
Os4CL1	0.15 ±0.01	0.88 ±0.06	0.14 ±0.00	0.05 ±0.01	0.05 ±0.00
Os4CL3	2.67 ±0.44	116.10 ±7.72	113.84 ±3.20	49.79 ±1.73	54.49 ±2.08
Os4CL5	0.05 ±0.00	0.33 ±0.02	0.25 ±0.02	0.10 ±0.01	0.01 ±0.00
OsC3H	5.53 ±0.04	40.06 ±1.00	21.09 ±1.16	8.46 ±0.15	9.90 ±0.39
OsC4H2	0.01 ±0.00	0.02 ±0.00	0.04 ±0.00	0.01 ±0.00	0.01 ±0.00
OsCAD1	1.32 ±0.08	14.03 ±1.70	7.24 ±0.44	2.84 ±0.01	6.19 ±0.39
OsCAD2	2.94 ±0.09	5.86 ±0.64	4.41 ±0.04	0.76 ±0.05	1.25 ±0.07
OsCAD8A	0.02 ±0.00	0.00 ±0.00	0.16 ±0.00	0.01 ±0.00	0.01 ±0.00
OsCAD8C	0.34 ±0.02	0.86 ±0.15	0.51 ±0.00	0.11 ±0.01	0.67 ±0.06
OsCCoAOMT1	3.20 ±0.15	8.72 ±0.26	5.43 ±0.61	1.73 ±0.05	3.74 ±0.13
OsCCoAOMT5	18.70 ±0.85	23.31 ±3.08	21.87 ±0.53	13.12 ±0.09	2.25 ±0.13
OsCCR1	0.00 ±0.00	0.04 ±0.00	0.04 ±0.00	7.05 ±0.29	0.10 ±0.00
OsCCR2	0.02 ±0.00	0.15 ±0.01	0.16 ±0.02	0.02 ±0.00	0.02 ±0.00
OsCCR3	0.00 ±0.00	0.00 ±0.00	0.00 ±0.00	0.00 ±0.00	0.00 ±0.00
OsCCR8	3.32 ±0.16	3.96 ±0.07	4.30 ±0.28	0.73 ±0.05	0.02 ±0.00
OsCOMT	52.25 ±1.04	140.51 ±5.39	54.55 ±3.83	7.13 ±0.26	17.09 ±0.95
OsCOMTL4	1.47 ±0.05	3.65 ±0.41	1.17 ±0.10	0.39 ±0.01	1.87 ±0.07
OsCOMTL6	0.03 ±0.00	0.02 ±0.00	0.02 ±0.00	0.01 ±0.00	0.09 ±0.00
OsF5H1	0.00 ±0.00	0.00 ±0.00	0.00 ±0.00	0.00 ±0.00	0.00 ±0.00
OsF5H2	0.00 ±0.00	0.00 ±0.00	0.00 ±0.00	0.00 ±0.00	0.00 ±0.00
OsF5H3	0.00 ±0.00	0.00 ±0.00	0.00 ±0.00	0.00 ±0.00	0.00 ±0.00
OsHCT1	11.17 ±0.59	24.91 ±1.36	7.96 ±1.14	4.13 ±0.23	8.72 ±0.71
OsHCT2	0.00 ±0.00	0.00 ±0.00	0.00 ±0.00	0.00 ±0.00	0.00 ±0.00
OsHCT3	0.00 ±0.00	0.00 ±0.00	0.00 ±0.00	0.00 ±0.00	0.00 ±0.00
OsPAL2	0.15 ±0.00	0.99 ±0.06	0.48 ±0.04	0.21 ±0.00	0.47 ±0.05
OsPAL8	0.45 ±0.03	5.83 ±0.61	11.07 ±1.47	8.27 ±0.07	0.90 ±0.05
OsPAL9	0.01 ±0.00	0.25 ±0.03	0.33 ±0.01	0.11 ±0.00	0.00 ±0.00

Table S8. The qRT-PCR gene expressions of 27 genes in the mutant C6.

Genes	I	II	III	IV	V
Os4CL1	0.13 ±0.01	0.37 ±0.03	0.54 ±0.03	0.13 ±0.01	0.06 ±0.00
Os4CL3	62.02 ±2.66	78.74 ±8.18	139.64 ±18.51	102.12 ±9.00	13.73 ±1.93
Os4CL5	0.51 ±0.04	0.70 ±0.03	0.35 ±0.01	0.57 ±0.05	0.01 ±0.00
OsC3H	28.73 ±0.91	24.56 ±3.78	30.70 ±2.44	19.63 ±2.40	3.73 ±0.42
OsC4H2	0.01 ±0.00	0.01 ±0.00	0.02 ±0.00	0.02 ±0.00	0.00 ±0.00
OsCAD1	6.92 ±0.78	2.80 ±0.18	5.01 ±0.28	5.42 ±0.66	2.82 ±0.36
OsCAD2	2.47 ±0.17	3.30 ±0.22	5.38 ±0.21	3.10 ±0.27	0.41 ±0.02
OsCAD8A	0.00 ±0.00	0.00 ±0.00	0.02 ±0.00	0.02 ±0.00	0.05 ±0.01
OsCAD8C	1.21 ±0.13	0.54 ±0.05	0.86 ±0.03	0.37 ±0.01	0.41 ±0.03
OsCCoAOMT1	6.01 ±0.32	3.74 ±0.51	11.18 ±0.78	4.84 ±0.61	2.06 ±0.32

OsCCoAOMT5	16.58 ±0.81	11.11 ±0.85	22.18 ±0.93	14.68 ±0.92	1.12 ±0.09
OsCCR1	0.01 ±0.00	0.02 ±0.00	0.08 ±0.01	0.06 ±0.01	0.03 ±0.00
OsCCR2	0.01 ±0.00	0.11 ±0.00	0.19 ±0.01	0.13 ±0.01	0.02 ±0.00
OsCCR3	0.00 ±0.00	0.00 ±0.00	0.00 ±0.00	0.01 ±0.00	0.00 ±0.00
OsCCR8	3.63 ±0.21	1.55 ±0.22	0.52 ±0.00	2.38 ±0.06	0.02 ±0.00
OsCOMT	53.85 ±4.62	51.73 ±6.86	97.39 ±10.07	32.61 ±3.73	7.47 ±0.30
OsCOMTL4	6.17 ±0.60	1.46 ±0.01	2.90 ±0.19	1.39 ±0.03	1.59 ±0.12
OsCOMTL6	0.64 ±0.03	0.17 ±0.02	0.62 ±0.05	0.73 ±0.08	1.11 ±0.02
OsF5H1	0.00 ±0.00	0.00 ±0.00	0.00 ±0.00	0.00 ±0.00	0.00 ±0.00
OsF5H2	0.00 ±0.00	0.00 ±0.00	0.00 ±0.00	0.00 ±0.00	0.00 ±0.00
OsF5H3	0.00 ±0.00	0.00 ±0.00	0.00 ±0.00	0.00 ±0.00	0.00 ±0.00
OsHCT1	10.50 ±1.09	12.49 ±1.75	19.24 ±1.34	8.86 ±1.07	5.77 ±0.69
OsHCT2	0.00 ±0.00	0.00 ±0.00	0.00 ±0.00	0.00 ±0.00	0.00 ±0.00
OsHCT3	0.00 ±0.00	0.00 ±0.00	0.00 ±0.00	0.00 ±0.00	0.00 ±0.00
OsPAL2	0.62 ±0.03	0.63 ±0.01	1.41 ±0.06	0.47 ±0.06	0.11 ±0.01
OsPAL8	15.44 ±0.80	22.92 ±3.04	23.56 ±0.97	22.10 ±2.87	0.82 ±0.01
OsPAL9	3.22 ±0.42	1.73 ±0.13	0.68 ±0.04	0.60 ±0.07	0.00 ±0.00

Table S9. The qRT-PCR gene expressions of 27 genes in the mutant C15.

Genes	I	II	III	IV	V
Os4CL1	0.10 ±0.01	0.34 ±0.02	2.64 ±0.19	0.47 ±0.04	0.03 ±0.00
Os4CL3	9.18 ±0.81	61.65 ±4.05	386.38 ±2.68	124.30 ±4.04	1.33 ±0.07
Os4CL5	0.01 ±0.00	0.05 ±0.01	1.21 ±0.10	0.29 ±0.02	0.01 ±0.00
OsC3H	3.50 ±0.23	10.44 ±1.49	63.07 ±3.74	20.29 ±1.27	1.38 ±0.07
OsC4H2	0.00 ±0.00	0.00 ±0.00	0.03 ±0.00	0.00 ±0.00	0.00 ±0.00
OsCAD1	0.48 ±0.02	1.19 ±0.06	8.81 ±0.57	3.96 ±0.22	2.41 ±0.11
OsCAD2	1.30 ±0.14	2.25 ±0.08	15.66 ±0.97	5.87 ±0.18	0.12 ±0.01
OsCAD8A	0.00 ±0.00	0.00 ±0.00	0.01 ±0.00	0.00 ±0.00	0.05 ±0.00
OsCAD8C	0.13 ±0.01	0.69 ±0.03	0.39 ±0.04	0.26 ±0.01	0.16 ±0.01
OsCCoAOMT1	0.37 ±0.03	1.42 ±0.05	11.29 ±0.83	3.41 ±0.40	0.87 ±0.06
OsCCoAOMT5	2.38 ±0.16	5.09 ±0.05	38.50 ±2.94	9.38 ±1.35	0.39 ±0.01
OsCCR1	0.00 ±0.00	0.00 ±0.00	0.58 ±0.04	0.23 ±0.04	0.00 ±0.00
OsCCR2	0.01 ±0.00	0.03 ±0.00	0.64 ±0.04	0.08 ±0.01	0.00 ±0.00
OsCCR3	0.00 ±0.00	0.00 ±0.00	0.00 ±0.00	0.00 ±0.00	0.00 ±0.00
OsCCR8	2.24 ±0.15	0.02 ±0.00	0.08 ±0.00	0.03 ±0.00	0.00 ±0.00
OsCOMT	13.96 ±0.99	38.79 ±1.13	220.34 ±17.52	69.98 ±4.13	1.35 ±0.06
OsCOMTL4	0.47 ±0.06	2.16 ±0.11	1.99 ±0.16	2.10 ±0.09	0.87 ±0.08
OsCOMTL6	0.03 ±0.00	0.03 ±0.00	0.05 ±0.00	0.10 ±0.00	0.31 ±0.01
OsF5H1	0.00 ±0.00	0.00 ±0.00	0.02 ±0.00	0.00 ±0.00	0.00 ±0.00
OsF5H2	0.00 ±0.00	0.00 ±0.00	0.00 ±0.00	0.00 ±0.00	0.00 ±0.00
OsF5H3	0.00 ±0.00	0.00 ±0.00	0.00 ±0.00	0.00 ±0.00	0.00 ±0.00
OsHCT1	4.06 ±0.14	5.98 ±0.05	47.68 ±2.51	19.62 ±0.47	1.18 ±0.05
OsHCT2	0.00 ±0.00	0.00 ±0.00	0.01 ±0.00	0.00 ±0.00	0.00 ±0.00
OsHCT3	0.00 ±0.00	0.00 ±0.00	0.00 ±0.00	0.00 ±0.00	0.00 ±0.00

OsPAL2	0.12 ±0.01	0.22 ±0.03	5.16 ±0.17	0.64 ±0.03	0.00 ±0.00
OsPAL8	0.45 ±0.03	3.78 ±0.16	47.54 ±1.36	10.94 ±0.57	0.10 ±0.01
OsPAL9	0.01 ±0.00	0.37 ±0.02	0.88 ±0.10	0.20 ±0.03	0.00 ±0.00

Table S10. The qRT-PCR gene expressions of 27 genes in the mutant C17.

Genes	I	II	III	IV	V
Os4CL1	0.14 ±0.01	0.51 ±0.04	1.69 ±0.11	0.19 ±0.01	0.03 ±0.00
Os4CL3	18.00 ±1.38	58.06 ±3.89	107.35 ±11.48	37.47 ±3.31	77.74 ±0.82
Os4CL5	0.27 ±0.02	1.92 ±0.03	0.70 ±0.04	0.03 ±0.00	0.01 ±0.00
OsC3H	7.64 ±0.05	15.76 ±0.66	21.94 ±2.20	7.08 ±0.77	4.49 ±0.41
OsC4H2	0.00 ±0.00	0.02 ±0.00	0.02 ±0.00	0.00 ±0.00	0.00 ±0.00
OsCAD1	2.06 ±0.10	3.63 ±0.34	6.89 ±0.25	1.77 ±0.04	2.55 ±0.33
OsCAD2	1.33 ±0.13	3.54 ±0.29	6.86 ±0.64	1.76 ±0.17	1.80 ±0.22
OsCAD8A	0.00 ±0.00	0.00 ±0.00	0.01 ±0.00	0.00 ±0.00	0.00 ±0.00
OsCAD8C	0.47 ±0.01	0.77 ±0.06	1.37 ±0.07	0.22 ±0.02	0.06 ±0.00
OsCCoAOMT1	1.57 ±0.02	3.85 ±0.32	6.77 ±0.33	1.32 ±0.07	1.47 ±0.06
OsCCoAOMT5	5.90 ±0.46	19.16 ±1.48	16.88 ±0.44	1.12 ±0.12	5.08 ±0.44
OsCCR1	0.01 ±0.00	0.07 ±0.00	0.09 ±0.00	0.02 ±0.00	0.16 ±0.01
OsCCR2	0.07 ±0.00	0.29 ±0.03	0.20 ±0.00	0.00 ±0.00	0.02 ±0.00
OsCCR3	0.00 ±0.00	0.01 ±0.00	0.00 ±0.00	0.00 ±0.00	0.00 ±0.00
OsCCR8	5.83 ±0.23	5.29 ±0.21	1.25 ±0.10	0.26 ±0.01	0.30 ±0.03
OsCOMT	13.66 ±0.82	40.44 ±1.40	105.03 ±4.37	20.24 ±2.12	27.78 ±3.85
OsCOMTL4	1.10 ±0.01	1.58 ±0.10	3.67 ±0.09	1.69 ±0.17	0.56 ±0.07
OsCOMTL6	0.02 ±0.00	0.04 ±0.00	0.06 ±0.00	0.20 ±0.01	0.27 ±0.04
OsF5H1	0.00 ±0.00	0.00 ±0.00	0.02 ±0.00	0.00 ±0.00	0.00 ±0.00
OsF5H2	0.00 ±0.00	0.00 ±0.00	0.00 ±0.00	0.00 ±0.00	0.00 ±0.00
OsF5H3	0.00 ±0.00	0.00 ±0.00	0.00 ±0.00	0.00 ±0.00	0.00 ±0.00
OsHCT1	4.26 ±0.05	8.35 ±0.84	13.94 ±0.37	5.78 ±0.56	14.88 ±0.71
OsHCT2	0.00 ±0.00	0.00 ±0.00	0.00 ±0.00	0.00 ±0.00	0.00 ±0.00
OsHCT3	0.00 ±0.00	0.00 ±0.00	0.00 ±0.00	0.00 ±0.00	0.00 ±0.00
OsPAL2	0.17 ±0.01	0.66 ±0.06	0.94 ±0.03	0.10 ±0.01	0.18 ±0.01
OsPAL8	5.68 ±0.40	15.94 ±0.44	7.27 ±0.38	1.03 ±0.07	1.46 ±0.12
OsPAL9	0.92 ±0.07	1.89 ±0.15	0.59 ±0.02	0.05 ±0.00	0.00 ±0.00

Table S11. The qRT-PCR gene expressions of 27 genes in the mutant Y102.

Genes	I	II	III	IV	V
Os4CL1	0.10 ±0.00	0.10 ±0.01	0.06 ±0.00	0.05 ±0.00	0.01 ±0.00
Os4CL3	8.31 ±0.74	15.32 ±0.37	9.65 ±0.37	17.82 ±1.06	0.74 ±0.05
Os4CL5	0.12 ±0.00	0.12 ±0.01	0.17 ±0.01	0.49 ±0.04	0.00 ±0.00
OsC3H	16.76 ±1.45	13.34 ±0.42	48.41 ±4.17	44.16 ±4.18	8.29 ±0.37
OsC4H2	0.03 ±0.00	0.01 ±0.00	0.05 ±0.00	0.02 ±0.00	0.01 ±0.00
OsCAD1	5.87 ±0.12	4.14 ±0.14	5.99 ±0.36	8.69 ±0.48	3.00 ±0.40
OsCAD2	2.70 ±0.13	2.58 ±0.09	18.12 ±1.91	10.46 ±0.23	1.84 ±0.27
OsCAD8A	0.06 ±0.00	0.00 ±0.00	0.04 ±0.00	0.00 ±0.00	0.01 ±0.00
OsCAD8C	1.08 ±0.15	1.40 ±0.06	0.56 ±0.04	0.57 ±0.04	0.09 ±0.01
OsCCoAOMT1	5.50 ±0.31	5.16 ±0.13	14.69 ±1.03	15.92 ±1.13	3.03 ±0.47
OsCCoAOMT5	10.68 ±0.04	11.44 ±0.82	43.65 ±1.52	23.30 ±1.39	6.08 ±0.72
OsCCR1	0.01 ±0.00	0.03 ±0.00	0.72 ±0.02	1.10 ±0.08	0.29 ±0.03
OsCCR2	0.12 ±0.00	0.13 ±0.01	0.36 ±0.01	0.56 ±0.05	0.06 ±0.00
OsCCR3	0.00 ±0.00	0.00 ±0.00	0.00 ±0.00	0.00 ±0.00	0.00 ±0.00
OsCCR8	2.14 ±0.11	1.61 ±0.05	1.25 ±0.07	0.54 ±0.04	0.04 ±0.01
OsCOMT	77.09 ±4.05	53.57 ±4.00	220.95 ±6.24	274.24 ±18.23	36.37 ±3.50
OsCOMTL4	3.98 ±0.40	3.40 ±0.33	1.14 ±0.02	3.16 ±0.16	0.41 ±0.03
OsCOMTL6	0.26 ±0.01	0.11 ±0.00	0.11 ±0.01	1.09 ±0.15	0.28 ±0.02
OsF5H1	0.01 ±0.00	0.01 ±0.00	0.00 ±0.00	0.01 ±0.00	0.00 ±0.00
OsF5H2	0.00 ±0.00	0.00 ±0.00	0.00 ±0.00	0.00 ±0.00	0.00 ±0.00
OsF5H3	0.01 ±0.00	0.00 ±0.00	0.00 ±0.00	0.00 ±0.00	0.00 ±0.00
OsHCT1	6.73 ±0.31	8.62 ±0.28	41.41 ±2.30	37.75 ±1.97	21.33 ±2.64
OsHCT2	0.00 ±0.00	0.00 ±0.00	0.00 ±0.00	0.00 ±0.00	0.00 ±0.00
OsHCT3	0.00 ±0.00	0.00 ±0.00	0.00 ±0.00	0.00 ±0.00	0.00 ±0.00
OsPAL2	0.62 ±0.02	1.07 ±0.06	5.57 ±0.23	3.92 ±0.38	0.80 ±0.06
OsPAL8	4.31 ±0.39	5.31 ±0.14	45.90 ±0.37	53.08 ±4.08	7.99 ±0.97
OsPAL9	0.32 ±0.03	0.40 ±0.05	0.45 ±0.03	0.47 ±0.02	0.00 ±0.00
